# Social participation, universal health coverage and health security

**DOI:** 10.2471/BLT.21.286554

**Published:** 2021-12-01

**Authors:** Helen Clark, Justin Koonin, Gabriela Cuevas Barron

**Affiliations:** a Auckland, New Zealand.; b ACON Health Limited, 414 Elizabeth St, Surry Hills, NSW 2010, Australia.; c Mexico City, Mexico.

The coronavirus disease 2019 (COVID-19) pandemic response is a good example of the growing chasm between people and their governments. To fight the pandemic, policy-makers often took top-down decisions with little community input, although these decisions were far reaching and affected people at all levels.^1^ Leaders have often used a default mode of governance that is neither inclusive nor particularly diverse,^2^^,^^3^ highlighting the shortcomings of how health systems have traditionally been steered.

This paradigm must change in the direction of transparency and inclusion if we are to make good on our commitments to universal health coverage and health security. Calls for this change are not new, but many governments repeatedly fail to proactively listen to people’s needs, perspectives and expectations as part of their decision-making processes, let alone to do so in systematic and institutionalized ways. Furthermore, groups with less access to policy spaces such as women, children, adolescents, people with disabilities and low-income communities are rarely engaged.

In our experience, leaders’ reluctance to embrace a truly inclusive health governance approach is due to several factors. These factors include existing sociocultural power imbalances that prevent meaningful interaction between stakeholders and policy-makers’ persistent adoption of a predominantly biomedical health model.^4^ Another crucial barrier is the systematic lack of policy-maker capacities to create, manage, sustain and leverage long-term, institutionalized participatory processes.

The ability to convene people from diverse backgrounds, deal adequately with conflicts of interest, broker dialogue between people with differing views and – more challengingly – make sense out of sometimes chaotic input emanating from a participatory space, must be promoted. Such ability requires policy-makers to have specific skillsets that need to be fostered, valued and cultivated. This capacity gap is one that the World Health Organization seeks to fill with the recently released publication *Voice, agency, empowerment - handbook on social participation for universal health coverage*.^5^

The handbook is an online resource that provides guidance for policy-makers on how social participation can be undertaken. It includes chapters on how to create an enabling environment for participation; ensure balanced and transparent representation; strengthen policy-makers’ capacities for meaningful engagement; increase the uptake of participatory process results; design legal frameworks for participation; and sustain engagement over time.

The handbook presents the fundamental premise that, within a participatory process intended for policy-making, meaningful interaction is brought about when decision-makers make visible efforts to level power imbalances within the participatory space. When such balance is ensured, both more influential and less powerful stakeholders feel safe to express their views. Creating such a space is essential for meaningful participation. Doing so requires governments to recognize the added value that participation offers and to acknowledge their own role within the process. Leaders must also be willing and able to reflect on societal power relations, welcome opposing views, and deal with potent interest groups – while also working to channel the engagement towards workable solutions.

Many countries, however, engage with their populations in ways that are not systematic or planned. While such an approach may be appropriate for certain policy questions, it should be seen only as a complement to regular government interaction with the people they serve. Countries should therefore work to create capacities and skills within their health systems to ensure that long-term and functional, social participation mechanisms are embedded in decision-making processes. Ideally, these mechanisms should be anchored in a legal framework and be provided with an adequate and predictable budget. Stable and available funding is critical for ensuring debate, discussion and exchange as a regular part of the way the health sector works. 

Best-practice examples of participatory decision- and policy-making in health are Thailand’s National Health Assembly,^6^ Tunisia’s Societal Dialogue for Health^7^ and France’s 2018 *Etats Généraux de la Bioéthique*.^8^ Such initiatives usually involve combining different participatory spaces and opportunities for exchange that ensure a broad-based interaction between members of the public, civil society actors, community groups, professional associations, government cadres and others. The objective is, above all, to listen, understand and learn how to jointly find a solution, despite opposing views, interests and vantage points.

Social participation is at the centre of good governance, and governance is one of the key enabling or hindering factors to both universal health coverage and health security.^9^ Therefore, investment in such social participation mechanisms is needed, and must start with building government capacities to create, manage and sustain such mechanisms for more institutionalized engagement with people. A regular interface between the different actors of society must be embedded within health system operations in a transparent, rational and policy-oriented manner.
